# Prevalence of Internet Gaming Disorder and Its Association With Personality Traits and Gaming Characteristics Among Chinese Adolescent Gamers

**DOI:** 10.3389/fpsyt.2020.598585

**Published:** 2020-11-17

**Authors:** Zhenjiang Liao, Qiuping Huang, Shucai Huang, Linxiang Tan, Tianli Shao, Ting Fang, Xinxin Chen, Shuhong Lin, Jing Qi, Yi Cai, Hongxian Shen

**Affiliations:** ^1^National Clinical Research Center for Mental Disorders, Department of Psychiatry, The Second Xiangya Hospital of Central South University, Changsha, China; ^2^Hunan Key Laboratory of Psychiatry and Mental Health, Hunan Medical Center for Mental Health, Chinese National Technology Institute on Mental Disorders, Institute of Mental Health of Central South University, Changsha, China; ^3^Department of Psychiatry, The Fourth People's Hospital of Wuhu, Wuhu, China; ^4^Education Center for Mental Health, Central South University, Changsha, China; ^5^Department of Psychiatry, Brain Hospital of Hunan Province, Changsha, China; ^6^Department of Psychiatry, Comorbid Somatic Diseases, Shenzhen Kangning Hospital, Shenzhen, China

**Keywords:** internet gaming disorder (IGD), internet gaming use characteristics, personality traits (big five), big five model, Chinese adolescent gamers

## Abstract

**Background:** Internet gaming is extremely popular in China. However, some players overuse it, with negative outcomes. Knowing the prevalence rate and specific risk factors can provide a better understanding of the etiology of Internet Gaming Disorder (IGD). This study aimed to estimate the prevalence of IGD among Chinese adolescents and its association with their personality traits and Internet gaming characteristics.

**Methods:** A cross-sectional study design was utilized in this study. Participants were recruited from 34 provincial administrative districts in China and consisted of 6,379 adolescent game players aged 15–25 (males/females = 3,701/2,678, mean age: 19.35 ± 1.61). A self-report structured questionnaire containing questions regarding demographic information and Internet gaming use characteristics, the Video Gaming Dependency Scale, and the Chinese Big Five Inventory-brief version, was used in the study.

**Results:** The prevalence of IGD among Chinese adolescent game players was 17.0%. All participants were divided into the IGD group (males/females = 751/333, mean age: 19.74 ± 1.85) or the non-IGD group (males/females = 2,950/2,345, mean age: 19.27 ± 1.54). Specifically, twelve factors were significantly associated with IGD (*p* < 0.001), including neuroticism (β = 0.17), conscientiousness (β = −0.14), Internet gaming time per day (Hour) (β = 0.21), Internet gaming charge per month (Yuan) (β = 0.21), motive: escaping (β = 0.15), motive: sensation seeking (β = 0.13), motive: maintaining (β = 0.08), motive: coping (β = 0.06), having one or two long-term game partners (β = 0.06), male (β = 0.12), undergraduate and above (β = 0.04), and relationship status of couple (β = 0.04).

**Conclusion:** Our findings offer evidence with respect to the prevalence of IGD and its relationships with specific personality traits and Internet gaming characteristics in China. It is necessary for Chinese parents, teachers, and social workers to pay more attention to adolescents' personality traits of high neuroticism and low conscientiousness, long time and high expense they spend on game, as well as their motives.

## Introduction

Given the characteristics of Internet games (free access, unlimited use, strong interactivity, and instant rewards), immersion in Internet gaming has become increasingly common and an important part of daily activities for adolescents and adults (1). As of June 2019, there were 494 million Internet game users in China, accounting for 57.8% of all Internet users (854 million) (2). Most people play games healthily, which can provide benefits for users (3). However, some players show overuse and symptoms similar to those of substance addictions (4), which may result in negative outcomes such as depression, anxiety, academic dysfunction, poor sleep quality, exacerbating relationships with family and peers, and even substance use (5, 6).

As a non-substance addiction (7), like gambling disorder (8), Internet Gaming disorder (IGD) was enrolled in the Fifth Edition of the Diagnostic and Statistical Manual of Mental Disorders (DSM-5) in 2013, warranting further research (9). In 2018, the International Classification of Diseases (ICD-11) also considered including it as a disease (10). Due to the differences in measurement tools and cultural backgrounds, different prevalence rates of IGD were reported among young people. For example, prevalence in Europe was between 1.2 and 1.6% (11, 12), while in Asia it ranged from 1.6 to 18.4% (13–15). Some studies reported that the rates of possible IGD among adolescents in China ranged between 2.0 (15) and 17.0% (16). However, more research on epidemiological investigation based on large samples should be conducted to verify those figures.

IGD is not only related to the stimulation of the game itself but also linked to the personality traits of the players. Personality traits can be considered as determinants of one's behaviors (17), which are enduring dispositions characterized by individuals' unique adaptation to others, themselves, objects, and even to the whole environment. According to the diathesis-stress model of behavior, addictive disorders may result from specific personality traits (1, 17). Strong empirical evidence has indicated that some personality traits were linked to antisocial behaviors, violence (18), substance use (19), and gambling (20). For example, heightened extraversion may be a predictor of smoking and alcohol consumption (21). Smokers scored higher on neuroticism and lower on conscientiousness and agreeableness than non-smokers (19).

Despite some controversies (22), the Five-Factor model is the most common classification of personality characteristics (23). The Big-Five model includes five personality dimensions: extraversion (people who are sociable and talkative), agreeableness (well-mannered and soft-hearted), neuroticism (unstable and short-tempered), conscientiousness (organized and hardworking), and openness to experience (curious and original) (24). A recent meta-analysis review revealed all personality dimensions had a significant effect on Internet addiction, based on 12 different studies (25). Easygoing, outgoing, responsible, and more neurotic individuals who were less open to experience showed higher levels of Internet addiction. Furthermore, individuals showing high levels of high conscientiousness (persistence, reliability, and an orientation toward achievement) may better predict general and mental health states (26). Studies of personality and health have shown that neuroticism in particular can be a common risk factor for poor health (27). To date, there has been limited research on the relationship between personality traits and IGD (1, 28–30), and these studies assessed personality traits using different assessment tools. Therefore, it is hard to compare or synthesize the results of their findings. Most research suggested a positive correlation between neuroticism and IGD; however, results for other personality traits have been inconsistent.

To better understand the risk factors of IGD, it is equally crucial to comprehend the association between IGD and Internet gaming use characteristics. Some researchers demonstrated that Internet gaming characteristics were significantly correlated with IGD. For example, Rho et al. (29) found that many significant behavioral factors can predict IGD, such as weekday game time, money spent on gaming, game club membership status, and offline game club attendance. Middle school students who played games more than 3 h a day were identified as having higher degrees of gaming addiction than others (30). Over 75% of gamers played with guild members; compared to playing solo, playing with others may be more stimulating (28).

This study is the first to investigate the association of IGD with the Big-Five factors and Internet gaming characteristics in a large sample of Chinese adolescent game players. It provides empirical evidence for understanding the etiology of IGD.

## Materials and Methods

### Subjects

We conducted our study as an online survey in China in October and November 2019 via WeChat (a social app used by more than one billion people) based on the survey program Questionnaire Star (a free online questionnaire survey evaluation and voting platform). People could browse the questionaire page by scanning the quick response (QR) code. In the first page, there was a brief introduction on the aim of this investigation, and its voluntary, anonymity and confidentiality, as well as the notes for filling in the questions. At the bottom of this page, persons could choose “reject” to refuse participating this survey, or “accept” to switch to the next page and continue filling the queastionaire. Anyone with access to the Internet who understand Chinese could participate. As an incentive for completing the questionnaire, participants could receive feedback on their results, including personality characteristics, and suggestions for their gaming behavior.

Online participants comprised adolescents and young adults, aged 15–25 years, from 34 provincial administrative districts in China. Among 8,592 respondents, 6,379 Internet game users were identified. Those who never played Internet games (*n* = 1403) or had severe physical or mental disorders (*n* = 462) were excluded. Additionally, we removed exaggerated responses (e.g., selected the same option for all items, *n* = 319) and multiple responses from the same IP addresses (*n* = 29).

The Institutional Review Board of Second Xiangya Hospital of Central South University approved this study protocol. All subjects were fully informed about the purpose of this investigation and signed online informed consent.

### Procedures and Measures

We used self-administered composite questionnaires to obtain demographic information, gaming use characteristics, IGD, and personality traits. We conducted a preliminary study in a small sample of adolescents and finalized the questionnaire before starting the study.

Demographic information included age, gender, relationship status (dating/married vs. single), highest education, and family structure (having siblings or being an only child).

For gaming use characteristics, respondents reported the game device (mobile or computer) they most commonly used, the number of long-term game partners they had, their motives for gaming, and the amount of money (monthly) and time (daily) they spent gaming over the past 12 months.

IGD was assessed by the Chinese version of Video Gaming Dependency Scale (VGD-S), which was revised from the English version developed by Rehbein et al. (11), covering all nine criteria of the DSM-5. For each criterion, there were two items. Participants were asked to choose each of the 18 descriptive items they had experienced in the past 12 months on a four-point Likert scale ranging from 1 (*total disagreement*) to 4 (*full agreement*). A criterion was met if at least one of the two items was answered with 4 (*full agreement*). Participants who endorsed five or more of the nine criteria were classified with IGD. The VGD-S was evaluated in a sample of 11,003 German students, aged 13–18, demonstrating good reliability of Cronbach's α with 0.93 (11). The Chinese version of the VGD-S yielded an internal consistency of 0.94 in this research.

Personality traits were assessed by the 40-item brief version of the Chinese Big Five Inventory (CBF-PI-B) (31–33). Big-five personality traits were measured with eight items mapping each dimension: conscientiousness, extraversion, agreeableness, neuroticism, and openness. Responses were on a six-point Likert scale ranging from 1 (*strongly disagree*) to 6 (*strongly agree*). The CBF-PI-B demonstrates high construct validity and reliability, and the internal consistency coefficient was between 0.76 (agreeableness) and 0.81 (neuroticism), with an average of 0.79 (31). The retest coefficient of the 10-week interval ranged from 0.67 (agreeableness) to 0.81 (openness), with an average of 0.74 (31). In this study the CBF-PI-B yielded an internal consistency of 0.86.

### Statistical Analysis

Prevalence of IGD in the whole sample was calculated. We used chi-square tests and *t-*tests to compare demographics, Internet gaming use characteristics, and personality traits between the IGD group and the non-IGD group. In univariate analysis, via a backward stepwise entry, multiple linear regression was performed to determine risk factors related to IGD. The statistical significance level was set at *p* < 0.001 (two-sided). We used SPSS software version 23.0 package for all analyses.

## Results

In this study, a total of 6,379 adolescent game players were recruited from 34 provincial administrative districts in China. The average age of the 6,379 participants was 19.4 years [standard deviation [SD]:1.6, range:15–25], and 57.1% were male adolescents (males/females = 3,701/2,678). The average time spent on gaming was 1.8 h per day (SD:1.3) and the average amount of money spent on gaming was 63.9 yuan per month (SD:145.2). Table 1 displayed the detailed sociodemographic and Internet gaming characteristics of the participants.

**Table 1:** Socio-demographic and Internet gaming characteristics of participants and comparison between IGD group and Non-IGD group by variables.

**Variables**		**Total (N = 6,379)**	**IGD group (*n =* 1,084)**	**Non-IGD group (*n =* 5,295)**	**c^**2**^ or t**	***p***
Gender	Male	3,701	751	2,950	68.001	<0.001
	Female	2,678	333	2,345		
Age (years)		19.35 ± 1.61	19.74 ± 1.85	19.27 ± 1.54	−8.743	<0.001
Relationship status	Couple	135	83	52	48.483	<0.001
	Single	6,244	1001	5,243		
Highest education	Below	2,641	353	2,288	42.035	<0.001
	Above	3,738	731	3,007		
Family structure	having siblings	3,166	530	2,636	0.285	0.773
	being an only child	3,213	554	2,659		
**Gaming use characteristics**
Device	PC	2,008	685	1,323	608.93	<0.001
	Mobile	4,371	399	3,972		
Long-term game partners	none	2,485	192	2,293	739.886	<0.001
	3>N≥1	2,027	479	1,548		
	6>N≥3	1,144	261	883		
	N≥6	723	152	571		
Daily gaming time(hours)		1.83 ± 1.30	2.86 ± 1.81	1.62 ± 1.05	−30.664	<0.001
Money spent on gaming/month(yuan)		63.92 ± 145.19	179.35 ± 241.44	40.29 ± 100.92	−30.791	<0.001
**Motives**
Seeking	Yes	1,915	589	1,326	367.529	<0.001
	No	4,464	495	3,969		
Escaping	Yes	694	345	349	590.99	<0.001
	No	5,685	739	4,946		
Coping	Yes	2,656	593	2,063	20.942	<0.001
	No	3,723	491	3,232		
Passing time	Yes	4,275	657	3,618	24.259	<0.001
	No	2,104	427	1,677		
Making friends	Yes	1,163	267	896	55.259	<0.001
	No	5,216	817	4,399		
Maintaining	Yes	1,350	355	995	116.45	<0.001
	No	5,029	729	4,300		
**Personality traits**
Neuroticism		25.68 ± 7.93	29.63 ± 6.38	24.87 ± 7.98	−18.492	<0.001
Conscientiousness		33.16 ± 6.34	30.52 ± 5.40	33.70 ± 6.39	15.286	<0.001
Openness		33.27 ± 6.96	32.35 ± 6.17	33.45 ± 7.10	4.781	<0.001
Agreeableness		35.10 ± 6.11	32.88 ± 5.85	35.56 ± 6.06	13.332	<0.001
Extraversion		29.65 ± 7.14	28.58 ± 6.57	29.87 ± 7.24	5.445	<0.001

*Below, Below the undergraduate; Above, Undergraduate or above; Seeking, Sensation seeking; Escaping, Escaping reality; Coping, Coping negative emotion; Friends, Making friends; Maintaining, Maintaining status*.

According to the positive criteria, endorsement of five or more of the nine criteria, a total of 1,084 adolescents (17%) were classified with IGD. All participants were divided into the IGD group (males/females = 751/333, mean age: 19.74 ± 1.85) and non-IGD group (males/females = 2,950/2,345, mean age: 19.27 ± 1.54). Results from the Chi-square tests and *T-*test (Table 1) show that IGD groups were more likely to be male (*p* < 0.001), single (*p* < 0.001), undergraduate or above (*p* < 0.001), older (*p* < 0.001), use computers to play (*p* < 0.001), have one or two long-term game players (*p* < 0.001), play games for seeking (*p* < 0.001) and escaping (*p* < 0.001), and spend more time and money on gaming (*p* < 0.001). To control the effect of socio-demographic variables, sex, education and relationship status, personality traits and Internet gaming characteristics were included in a multiple linear regression. All variables included in the linear regression model did not show multicollinearity (VIF: 1.021–2.207), and the Durbin–Watson was 1.906, so assumptions of collinearity were not violated. The association between IGD and predictive variables were strongly relative. Together, all the predictive variables accounted for 38.7% of the variance in IGD scores and the explained variability was also significant, F _(18,6378)_ = 224.276, *p* < 0.001.

Multiple linear regression analysis showed that twelve factors were significantly associated with IGD, including neuroticism (β = 0.17, *p* < 0.001), conscientiousness (β = −0.14, *p* < 0.001), Internet gaming time per day (Hour) (β = 0.21, *p* < 0.001), Internet gaming charge per month (Yuan) (β = 0.21, *p* < 0.001), motive: escaping (β = 0.15, *p* < 0.001), motive: sensation seeking (β = 0.13, *p* < 0.001), motive: Maintaining(β = 0.08, *p* < 0.001), motive: coping (β = 0.06, *p* < 0.001), having one or two long-term game partners (β = 0.06, *p* < 0.001), male (β = 0.12, *p* < 0.001), education of undergraduate or above (β = 0.04, *p* < 0.001), and relationship status of couple (β = 0.04, *p* < 0.001) (see Table 2).

**Table 2:** Multivariable linear regression of factors associated with IGD.

	**B**	**SE B**	**β**	***P***	**VIF**
Neuroticism	0.053	0.003	0.172	<0.001	1.206
Conscientiousness	−0.050	0.005	−0.140	<0.001	1.812
Agreeableness	0.011	0.005	0.028	0.016	1.438
Openness	0.006	0.005	0.017	0.237	2.207
Extraversion	−0.011	0.004	−0.027	0.027	1.523
Device	−0.03	0.059	−0.005	0.639	1.173
Long-term game partner	0.329	0.052	0.062	<0.001	1.021
Money spent on gaming/month	0.003	0.000	0.206	<0.001	1.354
Gaming time/day	0.395	0.021	0.209	<0.001	1.298
Sensation seeking	0.675	0.056	0.126	<0.001	1.141
Escaping	1.179	0.084	0.149	<0.001	1.180
Coping negative emotion	0.321	0.051	0.064	<0.001	1.075
Passing time	0.026	0.052	0.005	0.623	1.042
Making friends	0.062	0.064	0.010	0.327	1.039
Maintaining	0.481	0.060	0.080	<0.001	1.049
Gender	0.580	0.054	0.116	<0.001	1.217
Highest education	0.191	0.050	0.038	<0.001	1.023
Relationship states	0.719	0.176	0.042	<0.001	1.098

*This analysis was based on the IGD group and the Non-IGD groups. B, Regression coefficient; SE, standard error; β, standardized β coefficient. Model: F (18) = 224.28, p <0.001, R^2^ = 0.39*.

## Discussion

Our study is one of few to determine the relationships between IGD, Internet gaming use characteristics, and Big-Five personality traits. This is the first investigation of the prevalence of IGD in a large sample (>6,000) of adolescent game players in China. We found that nearly 17.0% of Chinese adolescent game users reported IGD, which was significantly associated with gender, highest education, relationship status, Internet gaming use characteristics (money spent per month, time spent per day, and long-term game partners), motivation (escape, sensation seeking, maintaining, and coping) and personality traits (neuroticism, conscientiousness).

In this study, we found a 17% prevalence of IGD among Chinese adolescent gaming users, the exact same percentage as the Experts Consensus of Gaming Disorder in China (2019 Edition) (16), which is much higher than many other countries. Using the same tools as our study, Rehbein et al. (11), the authors of the VGD-S English version, conducted a state-representative survey consisting of 11,003 middle school students and reported a 1.2% prevalence of GD. Müller et al. (12) found an IGD prevalence of 1.6% in 12,938 adolescents from seven European countries. Other studies reported significant figures: between 0.3 and 1.0% in four international groups of 18,932 people (34); 1.3% in a national sample of 902 Dutch gamers (35); and 1.8% of 1,287 Australian adolescents (36). The higher rate of IGD in this study might be related to differences in sample characteristics (based on the general population or gamers), assessments of IGD, or cultural factors. These studies, conducted in different socio-cultural populations, suggest that China's IGD prevalence is more concerning. This may be a cultural difference. Western countries have more individualistic cultures than Asian countries (37, 38), where higher levels of individualism were significantly associated with lower levels of Internet addiction, manifested in an increased need for achievement, dominance, and autonomy, and a reduced need for affiliation (39). Considering the negative health consequences of IGD and the vast number of Chinese adolescents, a 17% prevalence suggests that adolescent IGD in China deserves preventive and clinical attention.

Certain sociodemographic variables were more significantly associated with IGD. Consistent with previous studies (40, 41), we found that being male was significantly related with IGD, possibly due to gender differences in online use. To illustrate, females mostly use online networking for chatting, sharing information, tightening communication, and visiting personal websites, whereas males mostly use the Internet to play games (42). Furthermore, certain temperamental characteristics of males such as high comorbidity of Attention Deficit Hyperactivity Disorder (ADHD), high levels of novelty seeking, and high levels of impulsivity may be responsible for higher rates of IGD. Research also indicated that roughly four in five Massively Multiplayer Online Roleplaying Game (MMORPG) players were males (28). Many games on the market are invented by males for males (43); gaming companies strategically target boys and men to increase their sales (44, 45), partly explaining the high rate of male gaming usage.

Unlike previous studies (29, 46, 47), we found that dating or married and highly educated young adults were more likely to have IGD. Rho et al. (29) found there was no significant differece on marital status and education between the IGD and concrol group. A clinical research showed that patients with IGD were more likely single (46). Being single was positively associated to addictive video gaming, which may be related with their loneliness (47). The opposite results in this study may due to the unbalanced relationship status (135 couple vs. 6244 single) of these participants, which influenced the outcomes. The results of *t-*test showed that the IGD group was older than the non-IGD group. It can be speculated that young adults and college students tend to have more autonomy and opportunities to use smart devices than younger students do. Therefore, due to their technological proficiency and lack of external supervision, they may be more prone to suffer from gaming disorders (14). More research is needed to verify these results.

In the current study, IGD was significantly associated with the personality traits of neuroticism and conscientiousness, which is consistent with previous reports (48–50). Our study indicated a significant relationship between neuroticism and IGD, which correlates to a previous finding that when highly neurotic individuals consider the real world threatening, they often turn to digital worlds, where they feel in control and safe (1). Since previous research has reported that high level of neuroticism is a predictor for smoking (51) and correlated with other mental disorders (52), it may represent a general health risk factor. Our results also suggested a significant relationship between low conscientiousness and IGD. Low conscientiousness indicates one has less commitment to personal goals, a tendency to be disorganized, and trouble organizing and following a schedule (53); therefore, the digital environment may be more attractive (1). Consequently, paying particular attention to adolescents with high neuroticism or low conscientiousness may help prevent IGD.

We found that personality traits like openness, agreeableness, and extraversion were not associated with IGD. However, several studies reported a slightly significant correlation between low openness and IGD (54), low agreeableness associated with Internet addiction (55), and IGD patients displaying lower extraversion than control groups and groups of pathological gamblers (1). For example, game players with low levels of openness are prone to stick to their gaming behavior rather than exploring new activities (1). Further studies are needed to provide more evidence.

Certain Internet gaming characteristics, like having one or two long-term game partners, significantly predicted IGD. For example, MMORPGs encourage gamers to team up with other players to progress (28), inspiring teamwork and interaction (56, 57). Generally, gamers with IGD were considered to be isolated and have not many social relationships in the real world, while they developed long-term relationships with their game partners, such as attending offline game clubs and having game club memberships (29).

Consistent with findings on risk factors of IGD in the general population, the motives of escaping reality, sensation seeking, and coping with negative emotions were significantly associated with IGD. Sensation seeking may provide a coping strategy for boredom (58); this is a positive reinforcement for IGD. Furthermore, the diversity of online games is a preferable alternative to dealing with negative emotions (59) that are often associated with high neuroticism. Players who enjoy role-playing games engage in gaming to relax and temporarily escape real life (60). Alternatively, coping with negative emotions such as fear of failure can be seen as negative reinforcement for IGD (44, 61, 62). Many studies have shown that the feeling-of-escape motive is most strongly associated with and most predictive of IGD (28, 44).

Gaming costs and daily gaming time were additional risk factors of IGD. Users with IGD spent more time (2.86 vs. 1.62 h per day) and more money (179.35 vs. 40.29 yuan per month) than the non-IGD group. Those scoring low in conscientiousness had difficulty controlling the amount of time and money they spent on gaming. Previous studies reported that spending large amounts of time and money was a predictor of IGD (29). Many gamers report difficulty in controlling gaming time. Rho et al. (63) also showed that money and time spent on gaming are risk factors for IGD. Zaheer Hussain found that if a player spends more time playing games online, the risk of being addicted increases (28); therefore, corresponding measures should be taken to prevent and manage IGD, such as strictly controlling gaming time and money spent.

This study had some limitations. First, using self-reported questionnaires instead of face-to-face structured interviews requires that more caution is taken when generalizing the findings. However, self-reported investigations completed online are thought to improve honesty levels (64). Second, this study was a cross-sectional survey, so findings regarding relationships may not be causal. However, personality traits are relatively stable in life, so interpreting differences in personality traits is reasonable (1). Third, we used short versions of the Big-Five scale; although the complete versions may be more reliable and detailed, the short version appears to be psychometrical and more helpful for a large-scale survey. Fourth, the sociodemographic characteristics of the non-IGD group did not exactly match those of the IGD group, possibly leading to confounding results.

## Conclusions

This study found significant associations between IGD and personality factors, Internet gaming characteristics in China. Given that China appears to have a higher IGD prevalence rate than other regions, it is necessary for Chinese parents, teachers, and social workers to pay more attention to the mental needs of adolescents in terms of personality traits and gaming use. A three-level prevention system is worth adopting to assist with general prevention, targeted prevention, and early detection and treatment (16). Future research should be conducted to explore how these factors influence IGD and examine the development of IGD.

## Data Availability Statement

All relevant data is contained within the article: The original contributions presented in the study are included in the article/supplementary material, further inquiries can be directed to the corresponding author/s.

## Ethics Statement

The studies involving human participants were reviewed and approved by The Institutional Review Board of Second Xiangya Hospital of Central South University. The patients/participants provided their written informed consent to participate in this study.

## Author Contributions

All authors made substantial contributions to this study. ZL, QH, and HS conceptualized and designed the research, wrote the first draft of the manuscript, and contributed to the final manuscript. TS, TF, and XC prepared the assessment tools. LT, SL, and JQ performed the experiments. SH and YC undertook the statistical analysis. All authors critically reviewed content and approved final version for publication.

## Conflict of Interest

The authors declare that the research was conducted in the absence of any commercial or financial relationships that could be construed as a potential conflict of interest.

## References

[1] Müller KW Beutel M Egloff B Wölfling K Investigating risk factors for Internet gaming disorder: a comparison of patients with addictive gaming, pathological gamblers and healthy controls regarding the big five personality traits. Eur Addict Res. (2014) 20:129–36. 10.1159/000355832 24247280

[2] Cnnic The 44th China Statistical Report on Internet Development. Beijing (2019). p. 23.

[3] Wilms IL Petersen A Vangkilde S. Intensive video gaming improves encoding speed to visual short-term memory in young male adults. Acta Psychol. (2013) 142:108–18. 10.1016/j.actpsy.2012.11.003 23261420

[4] Young KS Internet addiction: the emergence of a new clinical disorder. Cyberpsychol Behav. (1998) 1:237–44. 10.1089/cpb.1998.1.237

[5] Greydanus DE Greydanus MM. Internet use, misuse, and addiction in adolescents: current issues and challenges. Int J Adolesc Med Health. (2012) 24:283–9. 10.1515/ijamh.2012.041 23183727

[6] Huang Q Li Y Huang S Qi J Shao T Chen X. Smartphone use and sleep quality in Chinese college students: a preliminary study. Front Psychiatry. (2020) 11:352. 10.3389/fpsyt.2020.00352 32435208 PMC7218048

[7] Association AP Diagnostic and Statistical Manual of Mental Disorders (DSM-5®) American Psychiatric Pub, Arlington, VA: American Psychiatric Association Publishing (2013).

[8] Mann K Fauth-Buhler M Higuchi S Potenza MN Saunders JB. Pathological gambling: a behavioral addiction. World Psychiatry. (2016) 15:297–8. 10.1002/wps.20373 27717269 PMC5032511

[9] Petry NM Rehbein F Gentile DA Lemmens JS Rumpf HJ Mossle T. An international consensus for assessing internet gaming disorder using the new DSM-5 approach. Addiction. (2014) 109:1399–406. 10.1111/add.12457 24456155

[10] World Health Organization WHO Releases New International Classification of Diseases (ICD 11) (2018). Available online at: https://www.who.int/news/item/18-06-2018-who-releases-new-international-classification-of-diseases-(icd-11)

[11] Rehbein F Kliem S Baier D Mößle T Petry NM. Prevalence of internet gaming disorder in German adolescents: diagnostic contribution of the nine DSM-5 criteria in a state-wide representative sample. Addiction. (2015) 110:842–51. 10.1111/add.12849 25598040

[12] Müller KW Janikian M Dreier M Wölfling K Beutel M Tzavara C. Regular gaming behavior and internet gaming disorder in European adolescents: results from a cross-national representative survey of prevalence, predictors, and psychopathological correlates. Eur Child Adolesc Psychiatry. (2015) 24:565–74. 10.1007/s00787-014-0611-2 25189795

[13] King DL Chamberlain SR Carragher N Billieux J Stein D Mueller K Screening and assessment tools for gaming disorder: a comprehensive systematic review. Clin Psychol Rev. (2020) 77:101831 10.1016/j.cpr.2020.101831 32143109

[14] Chia DXY Ng CWL Kandasami G Seow MYL Choo CC Chew PKH. Prevalence of internet addiction and gaming disorders in Southeast Asia: a meta-analysis. Int J Environ Res Public Health. (2020) 17:2582. 10.3390/ijerph17072582 32283803 PMC7177828

[15] Mak KK Lai CM Watanabe H Kim DI Bahar N Ramos M. Epidemiology of internet behaviors and addiction among adolescents in six Asian countries. Cyberpsychol Behav Soc Netw. (2014) 17:720–8. 10.1089/cyber.2014.0139 25405785

[16] Xiang YT Jin Y Zhang L Li L Ungvari GS Ng CH. An overview of the expert consensus on the prevention and treatment of gaming disorder in China (2019 Edition). Neurosci Bull. (2020) 36:825–8. 10.1007/s12264-020-00475-w 32125603 PMC7340689

[17] Mccrae RR Costa PT Jr. Personality trait structure as a human universal. Am Psychol. (1997) 52:509. 10.1037/0003-066X.52.5.509 9145021

[18] Yu R Geddes JR Fazel S. Personality disorders, violence, and antisocial behavior: a systematic review and meta-regression analysis. J Pers Disord. (2012) 26:775–92. 10.1521/pedi.2012.26.5.775 23013345

[19] Terracciano A Costa PT Jr. Smoking and the five-factor model of personality. Addiction. (2004) 99:472–81. 10.1111/j.1360-0443.2004.00687.x 15049747 PMC2376761

[20] Miller JD Mackillop J Fortune EE Maples J Lance CE Campbell WK. Personality correlates of pathological gambling derived from big three and big five personalitymodels. Psychiatry Res. (2013) 206:50–5. 10.1016/j.psychres.2012.09.042 23078872 PMC3635075

[21] Paunonen SV. Big five factors of personality and replicated predictions of behavior. J Pers Soc Psychol. (2003) 84:411–24. 10.1037/0022-3514.84.2.411 12585813

[22] Block J. A contrarian view of the five-factor approach to personality description. Psychol Bull. (1995) 117:187–215. 10.1037/0033-2909.117.2.187 7724687

[23] Mccrae RR 5 Years of Progress: A Reply to Block. Elsevier (2001) 35:108–13. 10.1006/jrpe.2000.2294

[24] Mccrae RR John OP. An introduction to the five-factor model and its applications. J Pers. (1992) 60:175–215. 10.1111/j.1467-6494.1992.tb00970.x 1635039

[25] Kayiş AR Satici SA Yilmaz MF Simşek D Ceyhan E Bakioglu F Big five-personality trait and internet addiction: a meta-analytic review. Comput Hum Behav. (2016) 63:35–40. 10.1016/j.chb.2016.05.012

[26] Christensen AJ Ehlers SL Wiebe JS Moran PJ Raichle K Ferneyhough K. Patient personality and mortality: a 4-year prospective examination of chronic renal insufficiency. Health Psychol. (2002) 21:315–20. 10.1037//0278-6133.21.4.315 12090673

[27] Brickman AL Yount SE Blaney NT Rothberg ST De-Nour AK. Personality traits and long-term health status. The influence of neuroticism and conscientiousness on renal deterioration in type-1 diabetes. Psychosomatics. (1996) 37:459–68. 10.1016/S0033-3182(96)71534-7 8824126

[28] Hussain Z Griffiths MD Baguley T Online gaming addiction: classification, prediction and associated risk factors. Addict Res Theory. (2012) 20:359–71. 10.3109/16066359.2011.640442

[29] Rho MJ Lee H Lee TH Cho H Jung DJ Kim DJ. Risk factors for internet gaming disorder: psychological factors and internet gaming characteristics. Int J Environ Res Public Health. (2018) 15:40. 10.3390/ijerph15010040 29280953 PMC5800139

[30] Karaca S Karakoc A Can Gurkan O Onan N Unsal Barlas G. Investigation of the online game addiction level, sociodemographic characteristics and social anxiety as risk factors for online game addiction in middle school students. Commun Ment Health J. (2020) 56:830–8. 10.1007/s10597-019-00544-z 31907803

[31] Wang MC Dai XY Yao SQ Development of the Chinese big five personality inventory (CBF-PI) III: psychometric properties of CBF-PI brief version. Chin J Clin Psychol. (2011) 19:454–7. 10.16128/j.cnki.1005-3611.2011.04.004

[32] Wang MC Dai XY Yao SQ. Development of the Chinese big five personality inventory (CBF-PI) II: validity analysis. Chin J Clin Psychol. (2010) 18:687–90. 10.16128/j.cnki.1005-3611.2015.04.00131454383

[33] Wang MC Dai XY Yao SQ Development of Chinese big five personality inventory(CBF-PI): theoretical framework and relability analysis. Chin J Clin Psychol. (2010) 18:545–8. 10.16128/j.cnki.1005-3611.2015.03.001

[34] Przybylski AK Weinstein N Murayama K. Internet gaming disorder: investigating the clinical relevance of a new phenomenon. Am J Psychiatry. (2017) 174:230–6. 10.1176/appi.ajp.2016.16020224 27809571

[35] Haagsma MC Pieterse ME Peters O. The prevalence of problematic video gamers in the Netherlands. Cyberpsychol Behav Soc Netw. (2012) 15:162–8. 10.1089/cyber.2011.0248 22313358

[36] King DL Delfabbro PH Zwaans T Kaptsis D. Clinical features and axis I comorbidity of Australian adolescent pathological Internet and video game users. Aust N Z J Psychiatry. (2013) 47:1058–67. 10.1177/0004867413491159 23719181

[37] Oyserman D Lee SW. Does culture influence what and how we think? Effects of priming individualism and collectivism. Psychol Bull. (2008) 134:311. 10.1037/0033-2909.134.2.311 18298274

[38] Oyserman D Coon HM Kemmelmeier M. Rethinking individualism and collectivism: evaluation of theoretical assumptions and meta-analyses. Psychol Bull. (2002) 128:3–72. 10.1037/0033-2909.128.1.3 11843547

[39] Arpaci I Kesici S Baloglu M Individualism and internet addiction: the mediating role of psychological needs. Int Res. (2018) 28:293–314. 10.1108/IntR-11-2016-0353

[40] Hyun GJ Han DH Lee YS Kang KD Yoo SK Chung US Risk factors associated with online game addiction:a hierarchical model. Comput Hum Behav. (2015) 48:706–13. 10.1016/j.chb.2015.02.008

[41] Yang DJ Chiu JZ Chen YK Examining the social influence on college students for playing online game: gender differences and implications. Turkish Online J Educ Technol. (2011) 10:115–22.

[42] Gross EF Adolescent Internet use: what we expect, what teens report. J Appl Dev Psychol. (2004) 25:633–49. 10.1016/j.appdev.2004.09.005

[43] Spekman ML Konijn EA Roelofsma PH Griffiths MD Gaming addiction, definition and measurement: a large-scale empirical study. Comput Hum Behav. (2013) 29:2150–5. 10.1016/j.chb.2013.05.015

[44] Kuss DJ. Internet gaming addiction: current perspectives. Psychol Res Behav Manag. (2013) 6:125–37. 10.2147/PRBM.S39476 24255603 PMC3832462

[45] Vollmer C Randler C Horzum MB Ayas T Computer game addiction in adolescents and its relationship to chronotype and personality. Sage Open. (2014) 4:2158244013518054 10.1177/2158244013518054

[46] Mallorquí-Bagué N Fernández-Aranda F Lozano-Madrid M Granero R Mestre-Bach G Baño M. Internet gaming disorder and online gambling disorder: clinical and personality correlates. J Behav Addict. (2017) 6:669–77. 10.1556/2006.6.2017.078 29280393 PMC6034948

[47] Schou Andreassen C Billieux J Griffiths MD Kuss DJ Demetrovics Z Mazzoni E. The relationship between addictive use of social media and video games and symptoms of psychiatric disorders: a large-scale cross-sectional study. Psychol Addict Behav. (2016) 30:252–62. 10.1037/adb0000160 26999354

[48] Van Der Aa N Overbeek G Engels RC Scholte RH Meerkerk G-J Van Den Eijnden RJ. Daily and compulsive internet use and well-being in adolescence: a diathesis-stress model based on big five personality traits. J Youth Adolesc. (2009) 38:765. 10.1007/s10964-008-9298-3 19636779

[49] Yan W Li Y Sui N. The relationship between recent stressful life events, personality traits, perceived family functioning and internet addiction among college students. Stress Health. (2014) 30:3–11. 10.1002/smi.2490 23616371

[50] Yao MZ He J Ko DM Pang K. The influence of personality, parental behaviors, and self-esteem on Internet addiction: a study of Chinese college students. Cyberpsychol Behav Soc Netw. (2014) 17:104–10. 10.1089/cyber.2012.0710 24003966 PMC3924803

[51] Soldz S Vaillant GE The big five personality traits and the life course: a 45-year longitudinal study. J Res Pers. (1999) 33:208–32. 10.1006/jrpe.1999.2243

[52] Kotov R Gamez W Schmidt F Watson D Linking “big” personality traits to anxiety, depressive, and substance use disorders: a meta-analysis. Psychol Bull. (2010) 136:768 10.1037/a0020327 20804236

[53] Ross C Orr ES Sisic M Arseneault JM Simmering MG Orr RR Personality and motivations associated with facebook use. Comput Hum Behav. (2009) 25:578–86. 10.1016/j.chb.2008.12.024

[54] Wang CW Ho RT Chan CL Tse S Exploring personality characteristics of Chinese adolescents with internet-related addictive behaviors: trait differences for gaming addiction and social networking addiction. Addict Behav. (2015) 42:32–5. 10.1016/j.addbeh.2014.10.039 25462651

[55] Kuss DJ Griffiths MD Binder JF Internet addiction in students: prevalence and risk factors. Comput Hum Behav. (2013) 29:959–66. 10.1016/j.chb.2012.12.024

[56] Griffiths M A ‘components’ model of addiction within a biopsychosocial framework. J Subst Use. (2005) 10:191–7. 10.1080/14659890500114359

[57] Hussain Z Griffiths MD Gender swapping and socializing in cyberspace: an exploratory study. Cyberpsychol Behav. (2008) 11:47–53. 10.1089/cpb.2007.0020 18275312

[58] Chiu S-I Lee JZ Huang DH. Video game addiction in children and teenagers in Taiwan. CyberPsychol Behav. (2004) 7:571–81. 10.1089/cpb.2004.7.571 15667052

[59] Hinojosa JA Méndez-Bértolo C Pozo MA. Looking at emotional words is not the same as reading emotional words: behavioral and neural correlates. Psychophysiology. (2010) 47:748–57. 10.1111/j.1469-8986.2010.00982.x 20158677

[60] Yee N. Motivations for play in online games. Cyber Psychol Behav. (2006) 9:772–5. 10.1089/cpb.2006.9.772 17201605

[61] Bass PF III Gaming addiction when going online goes off-kilter. Contemp Pediatr. (2015) 32:16.

[62] Columb D Griffiths MD O'gara C. Online gaming and gaming disorder: more than just a trivial pursuit. Ir J Psychol Med. (2019) 1–7. 10.1017/ipm.2019.31 31366420

[63] Rho MJ Jeong JE Chun JW Cho H Jung DJ Choi IY. Predictors and patterns of problematic Internet game use using a decision tree model. J Behav Addict. (2016) 5:500–9. 10.1556/2006.5.2016.051 27499227 PMC5264417

[64] Wood RT Griffiths MD Eatough V. Online data collection from video game players: methodological issues. Cyberpsychol Behav. (2004) 7:511–8. 10.1089/cpb.2004.7.511 15667045

